# Early puberty: a review on its role as a risk factor for metabolic and mental disorders

**DOI:** 10.3389/fped.2024.1326864

**Published:** 2024-09-12

**Authors:** Yukun Sun, Haiyan Liu, Chunguang Mu, Peipei Liu, Changfu Hao, Yongjuan Xin

**Affiliations:** ^1^ Department of Child and Adolescent Health, School of Public Health, Zhengzhou University, Zhengzhou, Henan, China; ^2^ Department of Emergency Response,Tongren Center for Disease Control and Prevention, Tongren, Guizhou, China; ^3^ Clinical Systems Biology Laboratories, Department of Neurology, The First Affiliated Hospital of Zhengzhou University, Zhengzhou, Henan, China

**Keywords:** early puberty, obesity, diabetes, cardiovascular diseases, behavioral dysfunction, depression

## Abstract

Accumulating evidence indicates that there is a trend of early puberty onset in humans. The early timing of puberty has raised concerns due to its association with significant negative health outcomes. However, overall impact and potential risk of early puberty remain uncertain. In this study, we conducted a comprehensive review of existing epidemiological studies to gain insights into the long-term adverse health effects associated with early puberty. Our objective was to provide a consolidated summary of these outcomes at a population level by considering studies that encompass various indicators of puberty. In all, early puberty has been identified as a potential risk factor for various metabolic diseases, such as obesity, diabetes, cardiovascular diseases (CVD). Children who experience early puberty are more likely to have a higher body mass index (BMI) during adulthood, increasing their risk of obesity. Early puberty also has been found to be an independent risk factor for diabetes mellitus, including gestational diabetes mellitus (GDM) and type 2 diabetes mellitus (T2DM), as earlier onset of menarche in girls and voice breaking in boys is associated with a higher prevalence of T2DM. Furthermore, evidence suggests that early puberty may contribute to an elevated risk of CVD, including conditions like coronary heart disease (CHD), stroke, angina, and hypertension. In addition, adolescents who experience early puberty, particularly girls, are more likely to suffer from mental problems, such as behavioral dysfunction and depression. Notably, early puberty has a more significant impact on girls than boys. Further research should consider the underlying mechanisms and potential preventive measures.

## The trend of puberty onset

Puberty is a crucial phase in human development, marking the transition from childhood to adulthood and encompassing significant physical, psychological, and social changes. It also signifies the attainment of reproductive capacity, which is essential for the continuity of any species. Typically, puberty begins between the ages of 8–13 years in girls and 9–14 years in boys, lasting for several years (1). However, emerging evidence suggests a trend towards earlier puberty onset in humans (2). Over the past century, many countries have witnessed a decline in the age at which girls experience breast development, pubarche, and menarche. A meta-analysis revealed that the global trend in the age at breast development (thelarche) decreased by nearly three months per decade from 1977 to 2013 (3). In White American girls, the average age of breast development decreased from 10.8 years to 10.3 years from 1948 to 1988–1994, with similar trends observed in Black and Mexican American girls. However, studies from Denmark and British cohorts did not exhibit a consistent downward trend, instead showing fluctuating patterns with an overall decline in girls (4, 5). Studies from Turkish cohort shown that the median age at pubarche development decreased by 0.7 years between 1973 and 2009 in girls (6). The age at menarche in the United States (US) initially declined (7), then stabilized between the 1900s and 1950s, and subsequently showed another decline from the 1990s onwards (8). In contrast, countries like Korea, South Africa, Mexico, and Romania had experienced a continuous decrease in the age at menarche (9–13). Although these observations highlight the variations in the timing of puberty across different populations, they all shown the phenomena of the trend of early puberty.

Research on puberty onset and development in girls has indeed received more attention compared to boys, with a particular focus on the timing of menarche. Although there is fewer epidemiological evidence, studies have also indicated a trend of early puberty in boys. For instance, research has shown that the age at Tanner stage G2 of genital development in White American boys decreased from 11.5 years to 10.1 years from 1951 to 2001 (14). In Europe, the age decreased from 12 years to 11.5 years from 1965 to 1997, considering the attainment of a testicular size of 4 ml as the marker of puberty onset. A recent population-based study in Sweden reported an earlier age of peak height velocity by 1.5 months per decade, which is an indicator of puberty, from 1947 to 1996 (15). Moreover, large-scale studies have suggested that the age at voice breaking is considerably earlier than previously reported (16). Despite the fewer number of studies available, all the above evidence suggesting a downward trend in the age of puberty onset in boys. It is crucial to conduct further research to better understand the factors contributing to early puberty and potential implications of advancing puberty in both sexes. Understanding these trends can help inform preventive measures and interventions to mitigate any potential negative consequences associated with early puberty.

## Pubertal markers

Our study aimed to conduct a comprehensive review of available epidemiological studies to summarize the long-term negative health outcomes of early puberty at a population level. Given the significant impact on children and their families, the primary focus was on metabolic and mental disorders. To achieve this, the study integrated studies that encompassed various puberty markers. The characteristics of relevant markers for pubertal timing are summarized in Table 1. In girls,breast development, also known as “thelarche”, is regarded as the gold standard for predicting the onset of puberty (14). This change is typically observed through self-evaluation and evaluation by a healthcare professional through visual inspection and palpation, although it can be challenging to distinguish in obese girls (26). The timing of menarche has received more attention than the timing of other pubertal milestones. There were relatively fewer studies on indicators such as breast development, pubarche, and other markers compared with menarche due to the difficulty in defining clear boundaries in girls. Menarche becomes the most common marker of puberty and is usually self-reported, but it may be susceptible to reporting bias (3). For boys, Genital development at Tanner stage G2 is considered the standard for predicting male puberty onset (27). The first conscious ejaculation is considered the counterpart of menarche in girls (20). Voice breaking, a distinct event during late puberty, is easily observable and noninvasive (21). Additionally, age of peak height velocity is an accurate and precise marker of puberty timing, requiring frequent annual measurements (24).

**Table 1:** Characteristics of relevant markers for puberty.

Gender	Pubertal markers	Characteristics	Advantages	Limitations
Women	Breast development	The first external signs of puberty (4)	Breast palpation is the best way to distinguish pubertal from prepubertal girls (17)	Difficult to distinguish from fat tissue by palpation (17)
	Menarche	A late pubertal phenomenon (3)	Easy to avail, noninvasive and most common pubertal marker(3)	Recording self-reporting and susceptible to reporting bias (3)
Men	Testicular enlargement	The first external signs of puberty (4)	Testicular volume of 4 ml is the first recognizable indicator of pubertal onset (18)	Impractical to use in population studies (19)
	First conscious ejaculation	A late pubertal phenomenon (20)	Easy to avail, noninvasive (20)	Data concerning ejacularche are very scarce (20)
	Voice breaking	A distinct event during late puberty (21)	Easy to avail, noninvasive (21)	Recording self-reporting and susceptible to reporting bias (22)
	Age at peak height velocity	The timing and intensity varies among individuals (23)	Noninvasive, accurate and precise (24)	Measured annually or more frequently (24)
Both	Pubarche	Appeared in early adolescence (18)	Noninvasive (22)	Examined by professional endocrinologist or pediatrician (25), often does not correlate with breast/gonadal development

## Early puberty and metabolic diseases

Puberty plays an important role in the development of metabolic diseases, particularly due to the rapid increase in insulin resistance during this period. The increase in insulin resistance can raise the risk of developing T2DM. Hormonal changes during puberty can also contribute to excess weight gain, increasing the risk of obesity. Early puberty onset can lead to various physical and psychological issues and increase the likelihood of developing metabolic diseases such as obesity, diabetes and cardiovascular diseases (CVD).

## Obesity

Obesity has become a major public health concern, with a significant increase in prevalence and associated healthcare costs. According to the WHO, the European region has witnessed an epidemic rise in overweight and obesity, affecting nearly 60% of adults and one in three school-aged children by 2022 (28). Epidemiologists have predicted that the steady increase in life expectancy may soon come to an end if effective population-level interventions are not implemented to curb the prevalence of obesity (29). The relationship between obesity and early puberty is bidirectional. Not only can childhood obesity trigger early puberty, but early puberty also can lead to adult obesity, characterized by high adipose tissue and high BMI. In this review, our primary focus was on the outcomes of the early puberty.

Children who experience early puberty are more likely to have a higher BMI and a greater risk of obesity later in life, with this association being more pronounced in women than in men. These associations often persist into adulthood (Table 2). A meta-analysis found that girls with early menarche had a higher adult BMI by 0.34 kg/m^2^ compared to girls with menarche after the age of 12 (49). Similar associations have been observed in various countries. In UK, girls with early menarche and boys with early age at voice breaking were at a higher risk of adult obesity compared to those with an average age of puberty (30). Another prospective study found that earlier age at menarche and age at voice breaking predicted a greater adult BMI by 0.52 kg/m^2^ and 0.34 kg/m^2^, respectively (31). Additionally, earlier menarche in mothers can predict higher weight and BMI in their children (50). These relationships have been observed in United States and European girls (32–34). After adjusting for confounding factors, earlier age at menarche was associated with a 31% increase in the odds of obesity (BMI between 30 and 34.9 kg/m^2^) and 34% increase in the odds of super obesity (BMI greater than 35 kg/m^2^) (35). In Chinese girls, earlier menarche was associated with an increase in BMI by 0.19 kg/m^2^ and waist circumference by 0.38 cm (36). These associations remained statistically significant when pubertal height growth spurt and peak height velocity were used as pubertal markers, in Finland and China (37, 38), although waist circumference change was not observed in Finland. It is important to note that there is limited research on the association between age at puberty and obesity in boys, with only a few epidemiological studies supporting these findings.

**Table 2:** Studies of early puberty and metabolic diseases.

Reference	Outcome	Region/study/sample(men/women)	Puberty measure	Adjusted factors	Findings
(30)	Obesity,T2DM,angin, hypertension, breast cancer	Britain/UK Biobank/(197714/25003) 20–49 years old	Age at menarche, Age at voice breaking	Age, Socioeconomic position and adiposity^a^	ORs for obesity, T2DM, angina and hypertension: 1.82 (1.77–1.87), 1.25 (1.15–1.36), 1.54 (1.41–1.68), 1.13 (1.1–1.16) in girls [8–11 vs.13 years (Ref)]; 1.58 (1.50–1.66), 1.24 (1.11–1.37), 1.39 (1.25–1.54), 1.11 (1.06–1.17) in boys [“younger” vs. “average”(Ref)]; OR for breast cancer:1.13 (1.06–1.20) in girls [8–11 vs.13 years (Ref)]
(31)	Obesity, triglycerides	Britain/MRCNSHD/(1051/999) 53 years old	Age at menarche, Age at voice breaking	BMI at 7 years	Increased triglycerides in girls and BMI (0.52 kg/m^2^/year in girls and 0.34 kg/m^2^/year in boys)
(32)	Obesity,T2DM	Europe/EPIC-InterAct/28557 52.4 years old in girls	Age at menarche	Age, BMI^a^	Increased BMI (0.32 kg/m^2^/year); HR for T2D:1.64 (1.46–1.83) [8–11 vs.13 years (Ref)]
(33)	Obesity, glucose, IFG	America/CARDIA/2583 42–59 years old in girls	Age at menarche	BMI^a^	Increased BMI (0.88 kg/m^2^/year), glucose (0.34 ± 0.11 mg/dl/year) and incident of IFG
(34)	Obesity,T2DM	America/BioCycle/25318 to 44 years old in girls	Age at menarche	Age, race, education, exercise	Increased BMI (1.35 kg/m^2)^, insulin (15 μIU/ml), HOMA-IR (0.15 U) and HOMA-β (0.16 U)[≤12 vs.13 years (Ref)]
(35)	Obesity	America/Midlife Women's Health Study/748 45–54 years old in girls	Age at menarche	Race, menopausal status, education, marital status, weight at age 18, alcohol use, and cigarette smoking	Each year increase in age at menarche, the odds of obesity and super obesity decreased by 31% (OR: 0.69 (0.59–0.81) and 34% [OR: 0.66 (0.52–0.83)]
(36)	Obesity	China/CKB/264979 30–79 years old girls	Age at menarche	Age, education, income, smoking, alcohol, exercise and OC using	Increased BMI (0.19 kg/m^2^/year) and waist circumference (0.38 cm/year)
(37)	Obesity, fasting insulin, diastolic blood pressure, HDL cholesterol	Finland/NFBC1966 study/(2417/2641) 30 years old	Age at pubertal height growth spurt	Childhood BMI	Increased adult BMI, fasting insulin, diastolic blood pressure and decreased HDL cholesterol in both sexes
(38)	Overweight, obesity	China/(7729/5414) 17–18-y-old	Age of peak height velocity	Height	HRs:1.16 (1.03–1.30) in boys;1.45 (1.21–1.75) in girls [P25< vs. P25-P75 (Ref)]
(14)	Diabetes, hypercholesterolemia	Mexico/MNHS/30626 > 20 years old in girls	Age at menarche	BMI	RR for diabetes and hypercholesterolemia:0.95 (0.93–0.98),0.93 (0.90–0.95) per year later age at menarche
(39)	T2DM	Korea/KNHANES/4657 20–50 years old in girls	Age at menarche	Age and BMI	OR:2.52 (1.29–4.94) [<12 vs. ≥12 years (Ref)]
(40)	T2DM	China/Jinchang Cohort/16114 45.8 years old in girls	Age at menarche	BMI	OR: 1.60 (1.16–2.22) [≤12 VS. 15–16 years (Ref)]
(41)	T2DM	China/Kadoorie Biobank/270345 23 years old in girls	Age at menarche	BMI	HR: 0.98 (0.97, 1.00) per year
(42)	T2DM	Sweden/UK Biobank/30697 > 30 years old in boys	Age of peak height velocity	Childhood BMI	HRs for early T2DM and late T2DM were 1.24 (1.17–1.31) and 1.11 (1.05–1.17) per year later age of peak height velocity
(43)	CVD, CHD, stroke	Britain/UK Biobank/482000 40–69 years old in girls	Age at menarche	Age, smoking, diabetes and BMI	HRs for CVD, CHD and strike were 1.18, 1.05 and 1.17 [<12 vs. >12 years (Ref)]
(44)	CHD, cerebrovascular diseases, hypertension	Britain/Million Women/1217840 67.5 years old in girls	Age at menarche	Age, BMI, smoking, alcohol, exercise	RR for CHD is 1.27 (1.22–1.31) [age ≤10 vs.13 years (Ref)]; associations were weaker for cerebrovascular and hypertensive disease than for CHD.
(45)	Hypertension	Britain/UK Biobank/(167020/194174) 40–69 years old	Age at menarche, Age at voice breaking	Children BMI	OR:0.89 (0.84–0.95) per year delay age at menarche
(46)	Hypertension	China/7518 35–75 years old in girls	Age at menarche	/	OR:0.965 (0.935–0.995) per year younger age at menarche
(47)	Stroke	Japan/Ohasama study/1412 > 35 years old in girls	Age at menarche	Age, height, BMI, smoking, alcohol, parity, hormone replacement therapy, menopause, hypertension, diabetes, hyperolesterolemia and heart disease	HR:1.83 (1.04–3.22) [≤13 vs.15 years (Ref)]
(48)	MACE	America/WISE/648 63.7 years old in girls	Age at menarche	CVD risk factors	HR:4.53 (2.13–9.63) [≤10 vs.12 years (Ref)]

^a^ Where “Obesity” and “BMI” were the outcome, the adjusted models not included “Obesity” and “BMI”. T2DM, type 2 diabetes mellitus; OR, odds ratio; BMI, body mass index; HR, hazard ratio; IFG, impaired fasting glucose; HDL, high-density lipoprotein; CVD, cardiovascular diseases; CHD, coronary heart disease; RR, relative risk; MACE, major adverse cardiovascular events; y, year; Ref, reference.

All the above evidence clearly indicates that early puberty is a risk factor for obesity in later life. Studies have shown that the association between pubertal timing and adult obesity remains statistically significant even after adjusting for BMI at an early age (31). The study conducted on 5058 subjects found that earlier pubertal timing was associated with higher adult BMI in both sexes, even after adjusting for factors reflecting fetal and childhood growth, including childhood BMI (37). A prospective cohort study found that the inverse association between age at menarche and BMI and obesity in middle age is not explained by confounding due to early childhood BMI. After Adjusting for childhood BMI, the age-adjusted change in mean adult BMI per additional year of age at menarche was −0.57 (−0.71, −0.43) (51). Additionally, a follow-up study found that boys with early age of peak height velocity tended to have higher waist and hip circumference, even after adjusting for childhood adiposity (52). Further researches are needed to examine this relationship between early puberty and obesity while adjusting for BMI.

## Diabetes

Diabetes was the ninth leading cause of death worldwide in 2018 (53). The causes of diabetes are complex with multiple factors, including nutritional factors, sedentary lifestyle, and psychosocial factors. Many studies have investigated the relationship between pubertal timing and diabetes. The evidence consistently suggests that early puberty is an independent risk factor for diabetes mellitus, including GDM and T2DM (Table 2). A systematic review in 2020 found that later age at menarche was associated with a lower risk of T2DM/IGT risk (RR = 0.91 per year), even after adjusting for adult adiposity (54). Data from the UK Biobank study indicated that the prevalence of T2DM was higher in girls with earlier menarche with odds ratio of 1.25 and boys with earlier age at voice breaking with odds ratio of 1.24, even after adjusting for socioeconomic position and adiposity (age 40–69 years) (30). Similar associations have been observed in European, Korean and Mexican girls (13, 32, 39). American girls with earlier menarche showed increased blood glucose levels (0.34 ± 0.11 mg/dl) (33). Metabolic markers for T2DM, such as insulin, HOMA-IR and HOMA-β, also showed a negative relationship with age at menarche (34). Studies in two cohorts suggested that earlier menarche was associated with an increased risk of T2DM (40, 41). When age of peak height velocity was used as a marker of puberty, this association was statistically significant in Swedish men after adjusting for childhood BMI (42). A large prospective cohort study found that early pubertal timing was associated with higher adult BMI in both sexes after adjusting for childhood BMI (40). Two other large prospective cohort studies found that a younger age at menarche was associated with an increased risk of T2DM, after adjustment for potential confounders such as body figure using a 9-level figure drawing (55) at age 10 years and BMI at 18 years (56). Additionally, girls with earlier menarche have a higher risk of developing GDM, which can have adverse effects on pregnancy outcomes (57–59). The underlying mechanisms linking early puberty and diabetes are complex and may involve impaired glucose tolerance (38) and β-cell function (53). Further studies are needed to better understand these associations, and the potential mechanisms, which could have important implications for preventive and therapeutic strategies.

## Cardiovascular diseases

Cardiovascular disease (CVD) is a severe condition and the leading cause of death worldwide (60). Common risk factors for CVD include high blood pressure, smoking, overweight or obesity, diabetes, and high cholesterol levels. In recent years, numerous epidemiologic studies have analyzed the relationship between early puberty and CVD. Studies suggested that early puberty may increase the risk of CVD, even after adjusting for age and BMI (Table 2). In UK, women with early menarche had a higher risk of CVD, including CHD [age-adjusted hazard ratio (HR) = 1.16] and stroke (age-adjusted HR = 1.22), compared to normal developmental women (43). Consistently, the Million Women study found a 1.27-fold increased risk of CHD and 1.16-fold increased risk of cerebrovascular disease in women with early menarche (44). Angina was also negatively associated with age at menarche in girls and age at voice breaking in boys (30). The Women's Ischemia Syndrome Evaluation study reported a 4.53-fold higher risk of adverse CVD outcomes in women with early age at menarche (≤10 years) (48). Furthermore, a meta-analysis revealed that increase in age at menarche was associated with a 3% lower relative risk of all-cause mortality (61). When age at menarche and age at voice breaking were used as the pubertal markers, children with earlier onset of menarche or voice breaking were more likely to have hypertension in UK and China (30, 46–63). This association was also identified in a meta-analysis of 17 studies (64). These results remained statistically significant even after adjusting for adult BMI (44, 65). Moreover, a birth cohort study and a mendelian randomization study suggested that early puberty is inversely associated with hypertension independent of childhood BMI (45, 66). Early puberty has also been associated with higher risk of stroke, diastolic blood pressure, triglycerides, and decreased high-density lipoprotein cholesterol (33, 37, 47). When integrating data on cardiovascular disease and early puberty, these health metrics can prove to be immensely valuable for cardiologists, healthcare providers, and public health experts in their assessments and decision-making endeavors.

## Early puberty and mental disorders

Adolescence is distinguished by swift physical transformations and psychosexual growth. The convergence of rapid physical maturation alongside gradual psychological development in this phase can potentially give rise to aberrant thoughts and behaviors, culminating in a spectrum of psychiatric issues. Such challenges may stem from hormonal fluctuations, social and emotional maturation, and encounters with stressful circumstances.

## Behavioral dysfunction

Behavioral dysfunction encompasses various aspects, including substance misuse, antisocial behavior, delinquency, and dating abuse victimization. There is substantial evidence indicating that early puberty is a risk factor for behavioral dysfunction (Table 3). Substance abuse remains a significant concern for adolescent health in Western countries, and it is more prevalent among individuals who experience early puberty (79). Longitudinal studies conducted in North Carolina found that adolescents with early menarche were more likely to engage in substance misuse, such as cigarettes, alcohol, and marijuana compared to their peers (67, 68). Drug abuse in the early menarche group displayed higher levels of self-reported criminality, substance use problems, social isolation, early sexual behavior, and psychiatric problems. By young adulthood, most of these differences had attenuated, but they were more likely to be depressed in young adulthood compared to their counterparts. Early maturers were also more likely to have had many sexual partners (68). Substance misuse can leads to risky sexual behaviors and adolescent pregnancy, with long-term effects such as reduced educational attainment, potential single parenthood, and economic disadvantage later in life (80). The Add Health study conducted in United States revealed that early age at menarche (<11 years) was associated with a higher incidence [5% of 1 standard deviation (SD)] of antisocial behavior compared to those who reached menarche at the mean age of 12 years, and this association often persisted into early-middle adulthood (69). Similarly, self-reported criminal behavior and school bullying are also prevalent among adolescents with early menarche (68, 70). The 2013 Youth Risk Behavior Survey showed that 20.9% of female students and 10.4% of male students in the US reported experiencing physical violence from a dating partner in the past 12 months (81). Early pubertal development, particularly in girls, is a risk factor for dating abuse victimization, as evidenced by several cohort studies (71, 82). Youths who experience dating abuse are at an increased risk of depression, anxiety, eating disorders, substance use and suicidal behavior (83–85). It is important to be vigilant, particularly for girls who mature earlier than their peers, as they may be targeted by older partners and are more likely to experience dating abuse. However, the underlying mechanisms of these associations are not yet fully understood. The social context may play a crucial role in these relationships. Studies have shown that deviant peer groups, negative school experience, harsh parenting, and neighborhood disorganization contribute to the interactions between pubertal timing, criminal behavior, and social competence (86). In fact, a prospective panel study indicated that girls with precocious puberty had a higher likelihood of associating with deviant peer groups and being exposed to harsher parenting practices (87).

**Table 3:** Studies of early puberty and mental disorders.

Reference	Outcome	Region/study/sample(men/women)	Puberty measure	Adjusted factors	Findings
(67)	Substance use	North Carolina/Context Study/6892 11–17 years old	Perceived pubertal timing	/	Increased risk of substance uses recently
(68)	Self-reported criminality, substance use, social isolation, early sexual behavior	North Carolina/GSMS/1420 14–19 years old in girl	Self-reported Tanner stage, Age at menarche	/	Increased incidence of all problems
(69)	Antisocial behaviors, depression symptoms	America/Add Health/7802 11–34 years old in girls	Age at menarche	Race, father absence, socioeconomic indicators, household income-to-needs ratio and maternal education	Increased incidence of all problems in early-middle adulthood
(70)	School bullying	Europe and America/HBSC study/227443 13.64 years old in girls	Age at menarche	Weight, family structure and socioeconomic, diet quality, classmate support and survey cycle	ORs for occasional victimization, perpetration, frequent victimization and perpetration were 1.21, 1.19 1.35, 1.46 in adolescent [<11 vs. ≥11 years (Ref)]
(71)	ADA	America/Add Health/3870 13–17 years old in girls	Age at menarche	Age, race, parents’ marital status, income, number of relationships, self-esteem and antisocial behavior history	Advanced pubertal development was associated with more ADA
(72)	Depression	North Carolina/GSMS/630 9–16 years old in girls	Tanner stage IV(Breast development and pubarche)	BMI and obesity status	OR:5.8 (1.9–17.9) [<12 vs. ≥12 years (Ref)]
(73)	Depression	China/5795 12–15 years old in girls	Breast development	Age, socioeconomic position, secondhand smoke exposure, parental age, survey mode, childhood BMI	OR:0.83 (0.70–0.98) per year increase
(74)	Depression	Britain/Add Health/1260 11–21 years old in girls	Age at menarche	/	Increased more depressive symptoms
(75)	Distress, and externalizing and fear disorders	America/NCS-A study/4925. 13–17 years old in girls	Age at menarche	Age, income, race, parent marital status, BMI, and childhood adversity	Increased distress, fear and externalizing disorders
(76)	Self-harm	America/ALSPAC/4042 16–21 years old in girls	Age at menarche	Maternal education, material hardship, maternal depression, childhood sexual abuse, parental separation and BMI	OR:1.31 (1.04–1.64) [<11.5 vs. 11.5–13.8 years (Ref)]
(77)	Psychological distress	France/6366 18–25 years old in girls	Age at menarche	Age and ethnicity	Increased risk of psychological distress
(78)	Suicidal ideation	Korea/KYRBS/35000 12–17 years old in girls	Age at menarche	Age, perceived stress, and depressive symptoms	Suicidal ideation was more prevalence in girls with early menarche

OR, odds ratio; ADA, adolescent dating abuse; PDS, pubertal development scale; BMI, body mass index; y, year; Ref, reference.

## Depression

Depression has a significant impact on human's health and well-being, causing more disability-adjusted life years than any other condition (88). Many convincing studies have shown a negative associated between pubertal timing and the incidence of depression (Table 3). Two prospective studies conducted in North Carolina identified that early age at menarche, Tanner stage IV, and higher testosterone levels were significant predictors of a greater risk of depression (OR = 5.8, 95% CI = 1.9–17.9). These disorders can persist into adulthood (69, 72). In China, individuals with early onset of breast development had a higher risk of depression, but this association was moderated when puberty timing was assessed by genitalia development in boys. Meanwhile, an earlier age at onset of public hair development was unrelated to the incidence of depression in girls and boys (73). Data from 630 female twin and sibling pairs showed that the prevalence of depression symptoms was higher in individuals with genetic predispositions toward earlier menarche (74). However, these findings were less evident in boys, as only a few retrospective studies have suggested an association between depression and early puberty or less mature pubertal status in boys. In all, depression symptoms are more pronounced in girls with early puberty than in boys.

In addition, early puberty may increase the risk of bulimia nervosa, anxiety disorders, distress disorders, fear, excessive psychosomatic symptoms, and self-harm (75–77) (Table 3). Notably, studies using data from the Korean Youth Risk Behavior Web-based Survey reported that girls with early menarche were more likely to have suicidal ideation (78). There are several explanations for this increased risk of psychopathology. First, many brain changes occur during puberty, and brain function is influenced by changes in gonadal hormones (89). For example, the release of dopamine, which is linked to certain symptoms of depression, can be influenced by sex hormones (90). Additionally, changes in neural systems may underlie disruptions in sleep, concentration, appetite, and sensation-seeking, which may increase the risk of mental disorders (75). Alternatively, early appearance of secondary sexual characteristics, which differ from those of peers, may increase levels of psychosocial stress and traumatic experiences from peers and adults, ultimately increasing the risk of mental disorders. Poor lifestyles, such as alcoholism and smoking, contribute to early puberty and can also induce mental health problems later in life (91–93).

## Conclusions and outlook

Various factors can trigger early puberty, and its effects, both short-term and long-term, have significant implications for the human body. In our current study, we aim to explore the origins of several adult disorders, making it crucial to investigate the long-term effects of early puberty. While many studies focus on the short-term developmental effects of early puberty, our research delves into its enduring impact, specifically on metabolic and mental disorders. Based on all the above, early pubertal timing is a risk factor for health outcomes. Compared to individuals with expected pubertal timing, adolescents with early puberty are more inclined to develop metabolic diseases. Children with early puberty are more likely to have a higher adult BMI and a greater risk of obesity, Studies have suggested that early pubertal timing has a potential causal effect on the risk of CVD, including CHD, stroke, angina, and hypertension. Additionally, it is important to note that adolescents who experience early puberty are more susceptible to mental problems, such as behavioral dysfunction and depression. With emotional disorders being more prevalent among adolescents and adults, investigating the impact of early puberty on psychological disorders will be a critical concern in the future. Therefore, greater attention from pediatricians and psychologists is needed for adolescents with early puberty, and strategies to prevent early puberty may be necessary. We also acknowledge two limitations in the current research. First, there is limited evidence to determine the relationship between early puberty and health outcomes in men. Second, Puberty encompasses various markers, and the use of diverse indicators in numerous studies presents a challenge in achieving an impartial and thorough review. Establishing a gold standard, similar to the BMI in obesity research, would enhance the specificity of studying its impact on metabolic disorders and other conditions. Our understanding of the adverse effects of earlier appearance of pubertal events is still limited. Most of these remain at the population research stage. Further studies are needed to clarify the long-term negative health outcomes of early puberty in men and to uncover the molecular mechanisms underlying the health outcomes of early puberty. This is important for predicting the risk of diseases and developing timely and effective interventions.

## References

[1] Santos Dos NR Rodrigues JLG Bandeira MJ Anjos ALDS Araújo CFDS Adan LFF Manganese and lead exposure and early puberty onset in children living near a ferromanganese alloy plant. Int J Environ Res Public Health. (2022) 19(12):7158. 10.3390/ijerph19127158 35742410 PMC9222911

[2] Thomsen AML Ramlau-Hansen CH Olsen J Brix N Andersen AN Lunddorf LLH The influence of parental age on timing of puberty: a study in the Danish national birth cohort. Scand J Public Health. (2022) 50(5):629–37. 10.1177/14034948211019794 34058902

[3] Eckert-Lind C Busch AS Petersen JH Biro FM Butler G Bräuner EV Worldwide secular trends in age at pubertal onset assessed by breast development among girls: a systematic review and meta-analysis. JAMA Pediatr. (2020) 174(4):e195881. 10.1001/jamapediatrics.2019.5881 32040143 PMC7042934

[4] Juul A Teilmann G Scheike T Hertel NT Holm K Laursen EM Pubertal development in Danish children: comparison of recent European and US data. Int J Androl. (2006) 29(1):247–55. discussion 286–90. 10.1111/j.1365-2605.2005.00556.x 16466546

[5] Christensen KY Maisonet M Rubin C Holmes A Flanders WD Heron J Progression through puberty in girls enrolled in a contemporary British cohort. J Adolesc Health. (2010) 47(3):282–9. 10.1016/j.jadohealth.2010.02.005 20708568 PMC5578456

[6] Atay Z Turan S Guran T Furman A Bereket A. Puberty and influencing factors in schoolgirls living in Istanbul: end of the secular trend? Pediatrics. (2011) 128(1):e40–5. 10.1542/peds.2010-2267 21669888

[7] Wyshak G Frisch RE. Evidence for a secular trend in age of menarche. N Engl J Med. (1982) 306(17):1033–5. 10.1056/NEJM198204293061707 7062994

[8] Morris DH Jones ME Schoemaker MJ Ashworth A Swerdlow AJ. Secular trends in age at menarche in women in the UK born 1908–93: results from the breakthrough generations study. Paediatr Perinat Epidemiol. (2011) 25(4):394–400. 10.1111/j.1365-3016.2011.01202.x 21649682

[9] Cho GJ Park HT Shin JH Hur JY Kim YT Kim SH Age at menarche in a Korean population: secular trends and influencing factors. Eur J Pediatr. (2010) 169(1):89–94. 10.1007/s00431-009-0993-1 19504269

[10] Jones LL Griffiths PL Norris SA Pettifor JM Cameron N. Age at menarche and the evidence for a positive secular trend in urban South Africa. Am J Hum Biol. (2009) 21(1):130–2. 10.1002/ajhb.20836 18942702

[11] Pop RM Tenenboum A Pop M. Secular trends in height, body mass and mean menarche age in Romanian children and adolescents, 1936–2016. Int J Environ Res Public Health. (2021) 18(2):490. 10.3390/ijerph18020490 33435327 PMC7827462

[12] Ahn JH Lim SW Song BS Seo J Lee JA Kim DH Age at menarche in the Korean female: secular trends and relationship to adulthood body mass index. Ann Pediatr Endocrinol Metab. (2013) 18(2):60–4. 10.6065/apem.2013.18.2.60 24904853 PMC4027094

[13] Petersohn I Zarate-Ortiz AG Cepeda-Lopez AC Melse-Boonstra A. Time trends in age at menarche and related non-communicable disease risk during the 20th century in Mexico. Nutrients. (2019) 11(2):394. 10.3390/nu11020394 30781889 PMC6412794

[14] Euling SY Herman-Giddens ME Lee PA Selevan SG Juul A Sørensen TI Examination of US puberty-timing data from 1940 to 1994 for secular trends: panel findings. Pediatrics. (2008) 121(Suppl 3):S172–91. 10.1542/peds.2007-1813D 18245511

[15] Ohlsson C Bygdell M Celind J Sondén A Tidblad A Sävendahl L Secular trends in pubertal growth acceleration in Swedish boys born from 1947 to 1996. JAMA Pediatr. (2019) 173(9):860–5. 10.1001/jamapediatrics.2019.2315 31329245 PMC6647355

[16] Brix N Ernst A Lauridsen LLB Parner E Støvring H Olsen J Timing of puberty in boys and girls: a population-based study. Paediatr Perinat Epidemiol. (2019) 33(1):70–8. 10.1111/ppe.12507 30307620 PMC6378593

[17] Fugl L Hagen CP Mieritz MG Tinggaard J Fallentin E Main KM Glandular breast tissue volume by magnetic resonance imaging in 100 healthy peripubertal girls: evaluation of clinical tanner staging. Pediatr Res. (2016) 80(4):526–30. 10.1038/pr.2016.125 27384405

[18] Villamor E Jansen EC. Nutritional determinants of the timing of puberty. Annu Rev Public Health. (2016) 37:33–46. 10.1146/annurev-publhealth-031914-122606 26789387

[19] Abreu AP Kaiser UB. Pubertal development and regulation. Lancet Diabetes Endocrinol. (2016) 4(3):254–64. 10.1016/S2213-8587(15)00418-0 26852256 PMC5192018

[20] Tomova A Lalabonova C Robeva RN Kumanov PT. Timing of pubertal maturation according to the age at first conscious ejaculation. Andrologia. (2011) 43(3):163–6. 10.1111/j.1439-0272.2009.01037.x 21486428

[21] Ong KK Bann D Wills AK Ward K Adams JE Hardy R Timing of voice breaking in males associated with growth and weight gain across the life course. J Clin Endocrinol Metab. (2012) 97(8):2844–52. 10.1210/jc.2011-3445 22654120 PMC3579950

[22] Huang A Roth CL. The link between obesity and puberty: what is new? Curr Opin Pediatr. (2021) 33(4):449–57. 10.1097/MOP.0000000000001035 34173790

[23] Nembidzane C Lesaoana, Monyeki KD Boateng A Makgae PJ. Using the SITAR method to estimate age at peak height velocity of children in rural South Africa: Ellisras longitudinal study. Children (Basel). (2020) 7(3):17. 10.3390/children7030017 32138295 PMC7140830

[24] Elhakeem A Frysz M Tilling K Tobias JH Lawlor DA. Association between age at puberty and bone accrual from 10 to 25 years of age. JAMA Netw Open. (2019) 2(8):e198918. 10.1001/jamanetworkopen.2019.8918 31397863 PMC6692837

[25] Liu Y Yu T Li X Pan D Lai X Chen Y Prevalence of precocious puberty among Chinese children: a school population-based study. Endocrine. (2021) 72(2):573–81. 10.1007/s12020-021-02630-3 33528762

[26] Magnus MC Guyatt AL Lawn RB Wyss AB Trajanoska K Küpers LK Identifying potential causal effects of age at menarche: a Mendelian randomization phenome-wide association study. BMC Med. (2020) 18(1):71. 10.1186/s12916-020-01515-y 32200763 PMC7087394

[27] Hamilton AS Mack TM. Puberty and genetic susceptibility to breast cancer in a case-control study in twins. Endocrinologist. (2004) 14(1):43. 10.1097/01.ten.0000092185.54927.bd 12788995

[28] The Lancet Public, H. Obesity prevention: changing perspectives. Lancet Public Health. (2023) 8(3):e161. 10.1016/S2468-2667(23)00033-6 36841553

[29] Olshansky SJ Passaro DJ Hershow RC Layden J Carnes BA Brody J A potential decline in life expectancy in the United States in the 21st century. N Engl J Med. (2005) 352(11):1138–45. 10.1056/NEJMsr043743 15784668

[30] Day FR Elks CE Murray A Ong KK Perry JR. Puberty timing associated with diabetes, cardiovascular disease and also diverse health outcomes in men and women: the UK biobank study. Sci Rep. (2015) 5:11208. 10.1038/srep11208 26084728 PMC4471670

[31] Pierce MB Kuh D Hardy R. Role of lifetime body mass index in the association between age at puberty and adult lipids: findings from men and women in a British birth cohort. Ann Epidemiol. (2010) 20(9):676–82. 10.1016/j.annepidem.2010.05.015 20696407 PMC2989433

[32] Elks CE Ong KK Scott RA van der Schouw YT Brand JS Wark PA Age at menarche and type 2 diabetes risk: the EPIC-interAct study. Diabetes Care. (2013) 36(11):3526–34. 10.2337/dc13-0446 24159179 PMC3816901

[33] Dreyfus J Jacobs DR Jr Mueller N Schreiner PJ Moran A Carnethon MR Age at menarche and cardiometabolic risk in adulthood: the coronary artery risk development in young adults study. J Pediatr. (2015) 167(2):344–52.e1. 10.1016/j.jpeds.2015.04.032 25962931 PMC4516565

[34] Chen L Zhang C Yeung E Ye A Mumford SL Wactawski-Wende J Age at menarche and metabolic markers for type 2 diabetes in premenopausal women: the BioCycle study. J Clin Endocrinol Metab. (2011) 96(6):E1007–12. 10.1210/jc.2010-2526 21470999 PMC3100755

[35] Gallicchio L Flaws JA Smith RL. Age at menarche, androgen concentrations, and midlife obesity: findings from the midlife women’s health study. Menopause. (2016) 23(11):1182–8. 10.1097/GME.0000000000000691 27433862 PMC5079777

[36] Yang L Li L Millwood IY Lewington S Guo Y Sherliker P Adiposity in relation to age at menarche and other reproductive factors among 300 000 Chinese women: findings from China kadoorie biobank study. Int J Epidemiol. (2017) 46(2):502–12. 10.1093/ije/dyw165 27524817 PMC5837303

[37] Widén E Silventoinen K Sovio U Ripatti S Cousminer DL Hartikainen AL Pubertal timing and growth influences cardiometabolic risk factors in adult males and females. Diabetes Care. (2012) 35(4):850–6. 10.2337/dc11-1365 22338106 PMC3308310

[38] Chen L Su B Zhang Y Ma T Liu J Yang Z Association between height growth patterns in puberty and stature in late adolescence: a longitudinal analysis in Chinese children and adolescents from 2006 to 2016. Front Endocrinol (Lausanne). (2022) 13:882840. 10.3389/fendo.2022.882840 35937794 PMC9354934

[39] Lim JS Lee HS Kim EY Yi KH Hwang JS. Early menarche increases the risk of type 2 diabetes in young and middle-aged Korean women. Diabet Med. (2015) 32(4):521–5. 10.1111/dme.12653 25441051

[40] Yang A Liu S Cheng N Pu H Dai M Ding J Reproductive factors and risk of type 2 diabetes in an occupational cohort of Chinese women. J Diabetes Complications. (2016) 30(7):1217–22. 10.1016/j.jdiacomp.2016.06.011 27345735

[41] Yang L Li L Peters SAE Clarke R Guo Y Chen Y Age at menarche and incidence of diabetes: a prospective study of 300,000 women in China. Am J Epidemiol. (2018) 187(2):190–8. 10.1093/aje/kwx219 28605451 PMC5860078

[42] Ohlsson C Bygdell M Nethander M Kindblom JM. Early puberty and risk for type 2 diabetes in men. Diabetologia. (2020) 63(6):1141–50. 10.1007/s00125-020-05121-8 32201902 PMC7228987

[43] Peters SA Woodward M. Women’s reproductive factors and incident cardiovascular disease in the UK biobank. Heart. (2018) 104(13):1069–75. 10.1136/heartjnl-2017-312289 29335253

[44] Canoy D Beral V Balkwill A Wright FL Kroll ME Reeves GK Age at menarche and risks of coronary heart and other vascular diseases in a large UK cohort. Circulation. (2015) 131(3):237–44. 10.1161/CIRCULATIONAHA.114.010070 25512444

[45] Chan II Kwok MK Schooling CM. Timing of pubertal development and midlife blood pressure in men and women: a Mendelian randomization study. J Clin Endocrinol Metab. (2022) 107(1):e386–93. 10.1210/clinem/dgab561 34343299

[46] Liu D Qin P Liu Y Sun X Li H Wu X Association of age at menarche with hypertension in rural Chinese women. J Hypertens. (2021) 39(3):476–83. 10.1097/HJH.0000000000002672 33044381

[47] Murakami K Metoki H Satoh M Asayama K Hosaka M Matsuda A Menstrual factors and stroke incidence in Japanese postmenopausal women: the ohasama study. Neuroepidemiology. (2016) 47(2):109–16. 10.1159/000452220 27820929

[48] Lee JJ Cook-Wiens G Johnson BD Braunstein GD Berga SL Stanczyk FZ Age at menarche and risk of cardiovascular disease outcomes: findings from the national heart lung and blood institute-sponsored women’s ischemia syndrome evaluation. J Am Heart Assoc. (2019) 8(12):e012406. 10.1161/JAHA.119.012406 31165670 PMC6645646

[49] Prentice P Viner RM. Pubertal timing and adult obesity and cardiometabolic risk in women and men: a systematic review and meta-analysis. Int J Obes (Lond). (2013) 37(8):1036–43. 10.1038/ijo.2012.177 23164700

[50] Ong KK Northstone K Wells JC Rubin C Ness AR Golding J Earlier mother’s age at menarche predicts rapid infancy growth and childhood obesity. PLoS Med. (2007) 4(4):e132. 10.1371/journal.pmed.0040132 17455989 PMC1876410

[51] Pierce MB Leon DA. Age at menarche and adult BMI in the Aberdeen children of the 1950s cohort study. Am J Clin Nutr. (2005) 82(4):733–9. 10.1093/ajcn/82.4.733 16210700

[52] Sandhu J Ben-Shlomo Y Cole TJ Holly J Davey Smith G. The impact of childhood body mass index on timing of puberty, adult stature and obesity: a follow-up study based on adolescent anthropometry recorded at Christ’s Hospital (1936–1964). Int J Obes (Lond). (2006) 30(1):14–22. 10.1038/sj.ijo.0803156 16344844

[53] Zheng Y Ley SH Hu FB. Global aetiology and epidemiology of type 2 diabetes mellitus and its complications. Nat Rev Endocrinol. (2018) 14(2):88–98. 10.1038/nrendo.2017.151 29219149

[54] Cheng TS Day FR Lakshman R Ong KK. Association of puberty timing with type 2 diabetes: a systematic review and meta-analysis. PLoS Med. (2020) 17(1):e1003017. 10.1371/journal.pmed.1003017 31905226 PMC6944335

[55] Baer HJ Colditz GA Rosner B Michels KB Rich-Edwards JW Hunter DJ Body fatness during childhood and adolescence and incidence of breast cancer in premenopausal women: a prospective cohort study. Breast Cancer Res. (2005) 7(3):R314–25. 10.1186/bcr998 15987426 PMC1143575

[56] He C Zhang C Hunter DJ Hankinson SE Buck Louis GM Hediger ML Age at menarche and risk of type 2 diabetes: results from 2 large prospective cohort studies. Am J Epidemiol. (2010) 171(3):334–44. 10.1093/aje/kwp372 20026580 PMC2842205

[57] Sun X Yang L Pan J Yang H Wu Y Chen Z Age at menarche and the risk of gestational diabetes mellitus: a systematic review and meta-analysis. Endocrine. (2018) 61(2):204–9. 10.1007/s12020-018-1581-9 29556913

[58] Petry CJ Ong KK Dunger DB. Age at menarche and the future risk of gestational diabetes: a systematic review and dose response meta-analysis. Acta Diabetol. (2018) 55(12):1209–19. 10.1007/s00592-018-1214-z 30159746 PMC6244847

[59] Qiu C Chen H Wen J Zhu P Lin F Huang B Associations between age at menarche and menopause with cardiovascular disease, diabetes, and osteoporosis in Chinese women. J Clin Endocrinol Metab. (2013) 98(4):1612–21. 10.1210/jc.2012-2919 23471979

[60] GBD 2016 Causes of Death Collaborators. Global, regional, and national age-sex specific mortality for 264 causes of death, 1980–2016: a systematic analysis for the global burden of disease study 2016. Lancet. (2017) 390(10100):1151–210. 10.1016/S0140-6736(17)32152-9 28919116 PMC5605883

[61] Charalampopoulos D McLoughlin A Elks CE Ong KK. Age at menarche and risks of all-cause and cardiovascular death: a systematic review and meta-analysis. Am J Epidemiol. (2014) 180(1):29–40. 10.1093/aje/kwu113 24920784 PMC4070937

[62] Werneck AO Oyeyemi AL Cyrino ES Ronque ERV Szwarcwald CL Coelho-E-Silva MJ Association between age at menarche and blood pressure in adulthood: is obesity an important mediator? Hypertens Res. (2018) 41(10):856–64. 10.1038/s41440-018-0079-4 30087421

[63] Global, regional, and national comparative risk assessment of 84 behavioural, environmental and occupational, and metabolic risks or clusters of risks for 195 countries and territories, 1990–2017: a systematic analysis for the global burden of disease study 2017. Lancet. (2018) 392(10159):1923–94. 10.1016/S0140-6736(18)32225-6 30496105 PMC6227755

[64] Bubach S De Mola CL Hardy R Dreyfus J Santos AC Horta BL. Early menarche and blood pressure in adulthood: systematic review and meta-analysis. J Public Health (Oxf). (2018) 40(3):476–84. 10.1093/pubmed/fdx118 28977577

[65] Lakshman R Forouhi NG Sharp SJ Luben R Bingham SA Khaw KT Early age at menarche associated with cardiovascular disease and mortality. J Clin Endocrinol Metab. (2009) 94(12):4953–60. 10.1210/jc.2009-1789 19880785

[66] Hardy R Kuh D Whincup PH Wadsworth ME. Age at puberty and adult blood pressure and body size in a British birth cohort study. J Hypertens. (2006) 24(1):59–66. 10.1097/01.hjh.0000198033.14848.93 16331102

[67] Cance JD Ennett ST Morgan-Lopez AA Foshee VA Talley AE. Perceived pubertal timing and recent substance use among adolescents: a longitudinal perspective. Addiction. (2013) 108(10):1845–54. 10.1111/add.12214 23680010 PMC4118468

[68] Copeland W Shanahan L Miller S Costello EJ Angold A Maughan B. Outcomes of early pubertal timing in young women: a prospective population-based study. Am J Psychiatry. (2010) 167(10):1218–25. 10.1176/appi.ajp.2010.09081190 20478880 PMC2992443

[69] Mendle J Ryan RM McKone KMP. Age at menarche, depression, and antisocial behavior in adulthood. Pediatrics. (2018) 141(1):e20171703. 10.1542/peds.2017-1703 29279324 PMC5744273

[70] Su Q Chen Z Li R Elgar FJ Liu Z Lian Q. Association between early menarche and school bullying. J Adolesc Health. (2018) 63(2):213–8. 10.1016/j.jadohealth.2018.02.008 29705494

[71] Chen FR Rothman EF Jaffee SR. Early puberty, friendship group characteristics, and dating abuse in US girls. Pediatrics. (2017) 139(6):e20162847. 10.1542/peds.2016-2847 28562261

[72] Copeland WE Worthman C Shanahan L Costello EJ Angold A. Early pubertal timing and testosterone associated with higher levels of adolescent depression in girls. J Am Acad Child Adolesc Psychiatry. (2019) 58(12):1197–206. 10.1016/j.jaac.2019.02.007 30768421 PMC6693999

[73] Wang H Lin SL Leung GM Schooling CM. Age at onset of puberty and adolescent depression: “children of 1997” birth cohort. Pediatrics. (2016) 137(6):e20153231. 10.1542/peds.2015-3231 27230766

[74] Mendle J Moore SR Briley DA Harden KP. Puberty, socioeconomic status, and depression in girls: evidence for gene×environment interactions. Clin Psychol Sci. (2016) 4(1):3–16. 10.1177/2167702614563598 32864196 PMC7450762

[75] Platt JM Colich NL McLaughlin KA Gary D Keyes KM. Transdiagnostic psychiatric disorder risk associated with early age of menarche: a latent modeling approach. Compr Psychiatry. (2017) 79:70–9. 10.1016/j.comppsych.2017.06.010 28757148 PMC5643227

[76] Roberts E Fraser A Gunnell D Joinson C Mars B. Timing of menarche and self-harm in adolescence and adulthood: a population-based cohort study. Psychol Med. (2020) 50(12):2010–8. 10.1017/S0033291719002095 31456538 PMC7525770

[77] Thériault J Otis J Hébert M Gurreri S Lambert J. Exploring the mediating and moderating roles of body-related concerns and social interactions on the association between early puberty and psychological distress in young adult women. Can J Public Health. (2019) 110(5):606–15. 10.17269/s41997-019-00213-4 31066011 PMC6964570

[78] Lee D Ahn IY Park CS Kim BJ Lee CS Cha B Early menarche as a risk factor for suicidal ideation in girls: the Korea youth risk behavior web-based survey. Psychiatry Res. (2020) 285:112706. 10.1016/j.psychres.2019.112706 31810746

[79] Ullsperger JM Nikolas MA. A meta-analytic review of the association between pubertal timing and psychopathology in adolescence: are there sex differences in risk? Psychol Bull. (2017) 143(9):903–38. 10.1037/bul0000106 28530427

[80] Deardorff J Gonzales NA Christopher FS Roosa MW Millsap RE. Early puberty and adolescent pregnancy: the influence of alcohol use. Pediatrics. (2005) 116(6):1451–6. 10.1542/peds.2005-0542 16322170

[81] Vagi KJ O'Malley Olsen E Basile KC Vivolo-Kantor AM. Teen dating violence (physical and sexual) among US high school students: findings from the 2013 national youth risk behavior survey. JAMA Pediatr. (2015) 169(5):474–82. 10.1001/jamapediatrics.2014.3577 25730143 PMC5858695

[82] Foster H Hagan J Brooks-Gunn J. Age, puberty, and exposure to intimate partner violence in adolescence. Ann N Y Acad Sci. (2004) 1036:151–66. 10.1196/annals.1330.009 15817736

[83] Exner-Cortens D Eckenrode J Rothman E. Longitudinal associations between teen dating violence victimization and adverse health outcomes. Pediatrics. (2013) 131(1):71–8. 10.1542/peds.2012-1029 23230075 PMC3529947

[84] Haynie DL Farhat T Brooks-Russell A Wang J Barbieri B Iannotti RJ. Dating violence perpetration and victimization among U.S. adolescents: prevalence, patterns, and associations with health complaints and substance use. J Adolesc Health. (2013) 53(2):194–201. 10.1016/j.jadohealth.2013.02.008 23664626 PMC3725188

[85] Foshee VA Reyes HL Gottfredson NC Chang LY Ennett ST. A longitudinal examination of psychological, behavioral, academic, and relationship consequences of dating abuse victimization among a primarily rural sample of adolescents. J Adolesc Health. (2013) 53(6):723–9. 10.1016/j.jadohealth.2013.06.016 23910572 PMC3838452

[86] Klopack ET Sutton TE Simons RL Simons LG. Disentangling the effects of boys’ pubertal timing: the importance of social context. J Youth Adolesc. (2020) 49(7):1393–405. 10.1007/s10964-019-01141-9 31587175 PMC9290439

[87] Klopack ET Simons RL Simons LG. Puberty and girls’ delinquency: a test of competing models explaining the relationship between pubertal development and delinquent behavior. Justice Q. (2020) 37(1):25–52. 10.1080/07418825.2018.1472291 32742073 PMC7394434

[88] Smith K. Mental health: a world of depression. Nature. (2014) 515(7526):181. 10.1038/515180a 25391942

[89] Schulz KM Sisk CL. The organizing actions of adolescent gonadal steroid hormones on brain and behavioral development. Neurosci Biobehav Rev. (2016) 70:148–58. 10.1016/j.neubiorev.2016.07.036 27497718 PMC5074860

[90] Taylor WD Zald DH Felger JC Christman S Claassen DO Horga G Influences of dopaminergic system dysfunction on late-life depression. Mol Psychiatry. (2022) 27(1):180–91. 10.1038/s41380-021-01265-0 34404915 PMC8850529

[91] Taylor GM Lindson N Farley A Leinberger-Jabari A Sawyer K Te Water Naudé R Smoking cessation for improving mental health. Cochrane Database Syst Rev. (2021) 3(3):Cd013522. 10.1002/14651858.CD013522.pub2 33687070 PMC8121093

[92] Brose LS Brown J McNeill A. Mental health and smoking cessation-a population survey in England. BMC Med. (2020) 18(1):161. 10.1186/s12916-020-01617-7 32580770 PMC7315517

[93] Luderer M Ramos Quiroga JA Faraone SV Zhang James Y Reif A. Alcohol use disorders and ADHD. Neurosci Biobehav Rev. (2021) 128:648–60. 10.1016/j.neubiorev.2021.07.010 34265320

